# The role of ANLN in malignant tumors: pathogenesis, treatment resistance and targeted strategies

**DOI:** 10.3389/fcell.2025.1739855

**Published:** 2026-01-07

**Authors:** YiXuan Wang, Yang Xiao, JieLin Yu, YuanHua Zou, Zhe Wang, XiangHong Yang

**Affiliations:** 1 Department of Pathology, Shengjing Hospital of China Medical University, Shenyang, China; 2 Department of Urology, General Hospital of Northern Theatre Command, China Medical University, Shenyang, China

**Keywords:** ANLN, biomarker, cancer, drug resistance, prognosis

## Abstract

ANLN is a highly conserved actin-binding protein that plays a critical role in cytokinesis by interacting with key cytoskeletal components such as actin, myosin, and RhoA. Increasing evidence shows that ANLN is aberrantly overexpressed in various cancers, including lung, breast, and liver cancers, and that its elevated expression is associated with enhanced tumor cell proliferation, migration, and invasion. Because of its central involvement in tumorigenesis and disease progression, ANLN has emerged as a promising prognostic biomarker and potential therapeutic target. Recent studies have demonstrated that ANLN contributes to resistance to chemotherapy, targeted therapy, and immunotherapy through multiple molecular mechanisms. This review provides a systematic overview of the physiological functions of ANLN, its roles in cancer initiation and progression, and its regulatory mechanisms in treatment resistance, offering biological insights into precision oncology and potential strategies for overcoming therapeutic resistance in cancer.

## 1 Introduction

Malignant tumors remain a major global health challenge, as the rising incidence and mortality rates of cancer continue to impose a heavy burden on populations worldwide. According to the 2022 cancer statistics in China, approximately 4.82 million new cancer cases and 2.57 million cancer-related deaths were reported, underscoring the substantial disease burden associated with malignancies (Zheng et al., 2024). Therefore, elucidating the molecular mechanisms underlying tumorigenesis and progression and identifying effective therapeutic targets are of great importance. Despite the widespread application of chemotherapy, targeted therapy, and immunotherapy, the emergence of drug resistance has evolved from a rare observation into a widespread and complex challenge that persists throughout cancer treatment, requiring urgent scientific and clinical attention.

Anillin(ANLN) is an evolutionarily conserved actin-binding protein that is ubiquitously expressed in eukaryotic cells. It primarily facilitates cytokinesis by interacting with actin filaments, microtubules, and septin cytoskeletal components, ensuring proper contractile ring formation and furrow ingression during cell division (Hohmann and Dehghani, 2019). Cytokinesis—the final stage of mitosis and meiosis that partitions cellular contents into daughter cells—is a fundamental biological process essential for maintaining genomic stability. Failure of this process can lead to binucleation and chromosomal instability, thereby promoting tumorigenesis (Piekny and Maddox, 2010). Recent studies have revealed that ANLN is aberrantly overexpressed in various cancers and promotes malignant progression by regulating oncogenic processes such as proliferation, migration, invasion, and drug resistance. Moreover, ANLN expression correlates with immune checkpoint molecules and the tumor microenvironment (Zhang et al., 2022a), suggesting its potential as both a prognostic biomarker and a therapeutic target. This review summarizes current insights into the physiological functions of ANLN, its mechanistic involvement in cancer development and progression, and its regulatory role in therapy resistance, aiming to provide a foundation for precision oncology and guide the development of novel therapeutic strategies.

## 2 The structure and function of ANLN

ANLN is an actin-binding protein that was first identified about 35 years ago through chromatographic analysis of actin filaments isolated from *Drosophila* embryos (Miller et al., 1989). The human *ANLN* gene is located on chromosome 7 and was initially recognized for its essential role in cytoplasmic division (Field and Alberts, 1995). Subsequent studies have revealed that ANLN functions extend beyond cell division to include cell migration, maintenance of cell polarity, and transcriptional regulation. Aberrant expression of ANLN has been closely associated with tumorigenesis and treatment resistance, making it an emerging focus in cancer drug resistance research.

### 2.1 The molecular structure of ANLN

**FIGURE 1:**
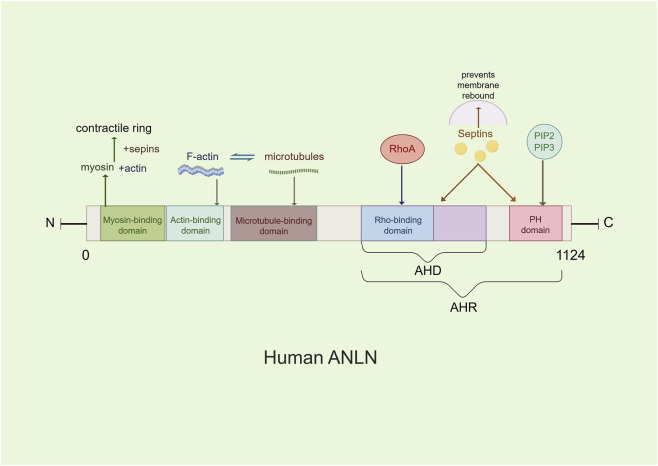
The main molecular structure model of the ANLN. ANLN contains several conserved domains, including a myosin II-binding domain, an actin-binding domain, an Anillin Homology Domain (AHD), and a pleckstrin homology domain(PH). These domains mediate interactions with cytoskeletal components and regulatory proteins involved in cytokinesis.

The N-terminal region of ANLN contains an actin-binding domain and a myosin-binding domain (Field and Alberts, 1995), which mediate nuclear localization during interphase, stabilize the actin cytoskeleton, and coordinate the assembly of the contractile ring with myosin II, thereby providing mechanical tension (Piekny and Glotzer, 2008). The C-terminal region includes an Anillin Homology Domain(AHD)—which interacts with the small GTPase RhoA—and a Pleckstrin Homology(PH) domain, collectively referred to as the Anillin Homology Region(AHR) (Figure 1). The AHD domain acts as a bridge between ANLN and RhoA, playing a crucial role in RhoA enrichment at the equatorial cortex, anchoring RhoA to the leading edge of pseudopodia, activating Rho-associated kinase(ROCK), driving myosin contraction, and localizing ANLN to the cleavage furrow (D’Avino, 2009; Oegema et al., 2000). The PH domain mediates cortical localization of ANLN during metaphase and its accumulation at the cleavage furrow (Piekny and Glotzer, 2008). ANLN recruits septin to form the septin-ANLN complex, establishing a physical barrier at the cleavage furrow. This complex promotes the unidirectional aggregation of contractile ring components, thereby stabilizing cell division (Maddox et al., 2007). Additionally, ANLN can directly bind to negatively charged membrane phospholipids, such as PIP2, through its PH domain and cryptic lipid-binding sites within the AHD, preventing membrane recoil in a septin-independent manner (Liu et al., 2012).

### 2.2 The biological functions of ANLN

ANLN is a central regulator of cytoskeletal dynamics, playing key roles in cytokinesis, cell junction maintenance, migration, and intranuclear transcriptional regulation. Through its N-terminal domain, ANLN binds to actin and myosin, anchors the contractile ring to the plasma membrane, ensures proper assembly and stability of the contractile ring, and maintains the fidelity of cell division (Kučera et al., 2021; Pollard and O’Shaughnessy, 2019). In addition, ANLN recruits septin proteins to form a barrier that prevents membrane rebound. Septin-actin polymers further recruit ANLN, forming a positive feedback loop that promotes contractile ring assembly (Maddox et al., 2007). During mitosis, ANLN interacts with actin filaments(F-actin) and microtubules to coordinate cell shape remodeling (Bareja et al., 2025; Beaudet et al., 2017; van Oostende Triplet et al., 2014). Moreover, phosphorylation of ANLN at S635 by ROCK enhances its binding to F-actin, stabilizing the contractile ring structure and inhibiting cell migration (Kim et al., 2017).

In non-dividing cells, ANLN regulates cell junctions and migration, enhancing resistance to mechanical stress, maintaining cell integrity and polarity, and promoting directional movement by anchoring the cytoskeletal network(e.g., actin cross-linking) (Piekny and Glotzer, 2008). It also modulates cell adhesion and junctional signaling; for instance, by inhibiting c-Jun N-terminal kinase (JNK) activity and stabilizing the perijunctional cytoskeleton, ANLN reinforces epithelial cell adhesion junctions (Wang D. et al., 2015). ANLN further promotes actin contractility by stabilizing active RhoA, thereby supporting tight junction repair and barrier maintenance (Craig et al., 2025). In cancer cells, ANLN participates in multiple signaling pathways, including activation of the RhoA/ROCK/myosin II axis, to enhance tumor cell migration (Wang D. et al., 2015). Mutations in the ANLN gene can disrupt podocyte junctions, leading to kidney disorders such as focal segmental glomerulosclerosis (Hall et al., 2018).

Recent evidence suggests that ANLN can also translocate into the nucleus to regulate gene transcription. Nuclear ANLN directly interacts with the large subunit of RNA polymerase II to form a transcriptional initiation complex, promoting transcription of target genes such as c-Myc and Cyclin D1 in the Wnt/β-catenin pathway and driving cell-cycle progression. This nuclear activity also enhances oxidoreductase function and increases transcriptional efficiency of differentiation-related genes (Cao et al., 2025).

## 3 The cross-cancer core driving mechanism of ANLN

**FIGURE 2:**
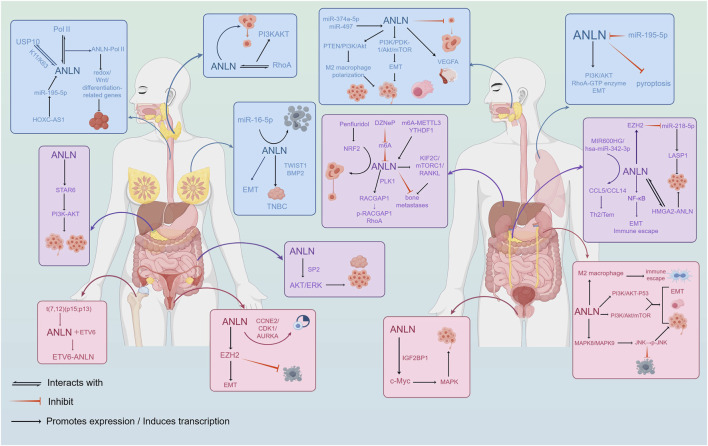
Biological functions and molecular pathway mechanisms of ANLN in tumor progression. ANLN regulates cell proliferation, migration, invasion, epithelial-mesenchymal transition(EMT), and cytokinesis through multiple signaling pathways, including RhoA/ROCK and PI3K/AKT.

Cancer arises from uncontrolled cell proliferation resulting from dysregulated signaling pathways. As a key regulatory factor in cytokinesis, ANLN is aberrantly expressed in a wide range of human cancers (Figure 2; Table 1). Pan-cancer analyses based on the TCGA database, along with multiple independent studies, have demonstrated that ANLN is overexpressed in diverse malignancies, including cancers of the lung, breast, liver(hepatocellular carcinoma), pancreas, and prostate, among others, suggesting its involvement in tumorigenesis and disease progression. Meanwhile, elevated ANLN expression is significantly associated with adverse clinicopathological features. High ANLN levels strongly correlate with increased tumor migration, invasion, metastasis, and poor clinical outcomes, making it a potential prognostic biomarker with broad predictive value across multiple cancer types. Notably, its oncogenic effects are not mediated through a single pathway but are driven by a complex network of interconnected core molecules.

**TABLE 1:** Tumor malignant progression mediated by ANLN and treatment resistance.

Cancer type	Expression	Clinical pathological features and prognosis	Associated pathways	Resistance mechanisms	References
Head & Neck(OSCC/NPC/HNSCC)	Upregulated	Poor prognosis	PI3K/PDK-1/Akt/mTOR in OSCC; ERK-MAPK→PD-L1 upregulation	Immune checkpoint resistance: ANLN↑→PD-L1↑, reduced anti-PD-1 efficacy	Guo et al. (2021), Wang B. et al. (2021), Zhu et al. (2025), Wang et al. (2025)
Esophageal squamous cell carcinoma	Upregulated	Poor prognosis	USP10-ANLN stabilization; nuclear ANLN-Pol II condensates; THZ1 disrupts ANLN-Pol II	—	Cao et al. (2023), Cao et al. (2025)
Lung cancer	Upregulated	Associated with age, gender, TNM/grade; poor OS/DFI/PFI	RHOA and PI3K/AKT activation; EMT involvement; pyroptosis suppression	Gemcitabine resistance *via* CYTOR/miR-125a-5p→ANLN; immunosuppressive microenvironment and therapy sensitivity patterns *via* CERS6-AS1/miR-424-5p→ANLN	Wang et al. (2023), Suzuki et al. (2005), Xu et al. (2019), Cao et al. (2024), Cao et al. (2024)
Breast cancer	Upregulated	Correlated with tumor size, high grade, HER2, Ki-67; poor OS/BCSS/RFS	EMT involvement; miR-16-5p→ANLN(cell cycle/apoptosis)	Doxorubicin resistance *via* ANLN–RhoA; PTX resistance *via* circ_0007331/miR-200b-3p→ANLN; Dox resistance *via* XIST/miR-200c-3p→ANLN; Lapatinib resistance *via* circ-MMP11/miR-153-3p→ANLN	Magnusson et al. (2016), Wang D. et al. (2020), Wang Z. et al. (2021), Wang F. et al. (2020), Zhang M. et al. (2020), Wu et al. (2021)
Hepatocellular carcinoma	Upregulated	Associated with AFP≥400 μg/L、tumor ≥8 cm; independent poor OS predictor	PLK1→RACGAP1→RhoA axis(cytokinesis fidelity); KEAP1–NRF2–MYC→ANLN(Penfluridol); m6A-METTL3/YTHDF1→ANLN→KIF2C/mTORC1/RANKL(bone metastasis)	Cholesterol–TAZ–TEAD2→ANLN/KIF23 axis drives resistance; reversible with pan-TEAD inhibitors or statins in combos	Wu et al. (2021), Zhang et al. (2021), Chen et al. (2022), Nguyen et al. (2024), Zheng et al. (2023), Saito et al. (2023)
Pancreatic cancer	Upregulated	Associated with size, differentiation, TNM, LN/distant metastasis; poor OS	EZH2/miR-218-5p→LASP1; NF-κB-linked glycolysis & EMT/immune escape; MIR600HG/miR-342-3p→ANLN(immune); HMGA2 binds ANLN promoter	Gemcitabine sensitivity restored by miR-217 *via* ANLN downregulation	Wang et al. (2019), Song et al. (2025), Qian et al. (2023), Guo et al. (2020), Panebianco et al. (2021)
Renal/Urinary(ccRCC/Bladder/UC)	Upregulated	Associated with advanced TNM/grade, metastasis; Higher grade, stage, LN+, vascular invasion; nuclear ANLN prognostic in UTUC	ccRCC: PI3K/Akt/mTOR; immunosuppressive M2 infiltration; Bladder: JNK(MAPK8/9) activation	—	Shan et al. (2025), Wei et al. (2019), Zeng et al. (2017), Wei et al. (2019), Zeng et al. (2017), Gao et al. (2024), Chen et al. (2023)
Colorectal cancer	Upregulated	High ANLN associated with invasion/growth; independent Poor prognosis	SP2-mediated transcription; AKT/ERK phosphorylation	—	Wang et al. (2016), Liu et al. (2022)
Prostate cancer	Upregulated	Higher T/N stage, Gleason, PSA; poor prognosis	IGF2BP1→c-Myc & MAPK signaling; VDRE/1,25-(OH)2D3-linked regulation of ANLN	—	Takayama et al. (2019), Yamamoto et al. (2024), Takayama et al. (2019), Yamamoto et al. (2024), Johnson et al. (2024)
Cervical cancer	Upregulated	Poor survival with high expression; ANLN in 6-gene prognostic model; Associated with LN metastasis network	EZH2-mediated EMT/apoptosis inhibition	—	Xia et al. (2018), Ding et al. (2023), Pan et al. (2022)

### 3.1 Cytoskeleton and cell cycle regulation

As a key regulator of cell division, ANLN promotes tumor cell proliferation and drives malignant progression by orchestrating cell-cycle progression and ensuring cytokinetic fidelity across multiple cancer types. ANLN facilitates aberrant cell proliferation by modulating the G2/M transition and upregulating critical cell-cycle proteins, including Cyclin B1 and Cyclin D1. In lung cancer, ANLN downregulation induces G0/G1 arrest and enhances apoptosis (Tomioka et al., 2025). Similarly, studies in bladder cancer show that ANLN knockdown triggers G2/M arrest and decreases Cyclin B1 and Cyclin D1 expression, thereby suppressing tumor growth (Zeng et al., 2017). In colon cancer, ANLN silencing inhibits proliferation, induces G0/G1 arrest, and markedly impairs tumorigenic potential *in vivo* (Liu et al., 2022). Moreover, miR-16-5p targets ANLN to accelerate the G2/M transition, shorten the overall cell-cycle duration, and promote apoptosis in breast cancer cells (Wang Z. et al., 2021). ANLN is also essential for mitotic fidelity, functioning to precisely regulate the assembly and stability of the contractile ring during cytokinesis. Notably, ANLN depletion increases polyploidy in hepatocytes, a process thought to confer resistance to carcinogenic insults while preserving genomic integrity without inducing regeneration defects or non-diploid risks—suggesting a potentially protective role in liver cancer development (Lin et al., 2020; Zhang et al., 2018). In glioma, ANLN interacts with phosphorylated myosin regulatory light chain(MRLC) mediated by STK17A, leading to defective cytokinesis and directly contributing to tumorigenesis (Chen et al., 2019). Additionally, as a downstream effector of RhoA GTPase, ANLN activates RhoA-mediated signaling to enhance actomyosin contractility, a mechanism recognized as essential for promoting lung cancer cell migration and invasion (Suzuki et al., 2005). In liver cancer, ANLN promotes PLK1-mediated phosphorylation of RACGAP1 and subsequent activation of RhoA, thereby ensuring mitotic accuracy, whereas ANLN inhibition disrupts mitosis and effectively suppresses tumor growth (Chen et al., 2022).

### 3.2 Cell migration and epithelial-mesenchymal transition(EMT)

Epithelial-mesenchymal transition(EMT) is a central mechanism driving tumor metastasis. ANLN enhances EMT, stabilizes the mesenchymal phenotype, maintains cancer stemness, and promotes cell migration. In lung, breast, cervical, and clear cell renal cell carcinomas, inhibition of ANLN leads to increased expression of epithelial markers such as E-cadherin and decreased expression of mesenchymal markers including N-cadherin and vimentin, resulting in significantly reduced migration and invasio (Pan et al., 2022; Shan et al., 2025; Wang D. et al., 2020; Xu et al., 2019). In triple-negative breast cancer, ANLN enhances tumor stemness *via* the TWIST1–BMP2 axis, coordinates EMT-related transcriptional programs, promotes extracellular matrix degradation, and consequently facilitates tumor initiation and progression (Maryam and Chin, 2021). In cervical cancer, ANLN activates EZH2 to drive EMT and inhibit apoptosis, thereby enhancing tumor cell migration (Pan et al., 2022). IIn oral squamous cell carcinoma, ANLN knockdown increases expression of epithelial markers such as cadherin-1(CDH1) and claudin-1(CLDN1), while reducing mesenchymal markers including vimentin, SNAIL1, and SNAIL2, leading to attenuated EMT (Wu et al., 2024). In pancreatic cancer, ANLN depletion downregulates multiple cell-adhesion-related genes, particularly LIM and SH3 domain protein 1(LASP1), thereby impairing migration, invasion, and tumorigenic capacity (Wu et al., 2024). Collectively, these findings establish ANLN as a critical regulator of cell adhesion remodeling and metastatic potential.

### 3.3 Activation of the key signaling pathway PI3K/AKT

Among the downstream signaling pathways regulated by ANLN, the PI3K/AKT pathway is the most consistently activated across cancer types. ANLN activates PI3K/AKT signaling to enhance proliferation and migration, inhibit apoptosis, and drive malignant progression. In oral cancer, ANLN activation leads to phosphorylation of PI3K, mTOR, AKT, and PDK-1, resulting in markedly elevated pathway activity that promotes tumor development (Wang B. et al., 2021). In anaplastic thyroid carcinoma, ANLN interacts with RhoA to activate the PI3K/AKT pathway and facilitate tumor progression (Yu et al., 2025). In lung cancer, ANLN enhances proliferation and suppresses apoptosis through AKT phosphorylation (Suzuki et al., 2005). ANLN also activates PI3K/AKT signaling by upregulating STRA6, thereby promoting proliferation and migration in gallbladder cancer (Zhu et al., 2024). In clear cell renal cell carcinoma(ccRCC), ANLN modulates the PI3K/AKT/mTOR axis to reinforce malignant cellular behavior (Gao et al., 2024). Through activation of PI3K/AKT signaling, ANLN coordinates downstream transcriptional programs that regulate essential processes such as cell growth, metabolic adaptation, and survival.

## 4 The expression and function of ANLN in different cancers

### 4.1 The role and mechanism of ANLN in head and neck cancer

ANLN is significantly overexpressed in oral squamous cell carcinoma(OSCC), nasopharyngeal carcinoma(NPC), and head and neck squamous cell carcinoma(HNSCC). Its high expression is strongly correlated with shorter survival in patients with these cancers (Guo et al., 2021; Wang B. et al., 2021; Zhu et al., 2025). The highly expressed ANLN is positively correlated with the advanced clinical stage of NPC (Zhu et al., 2025).

ANLN contributes to the initiation and progression of head and neck cancers by modulating vascular activity and promoting tumor-associated macrophage polarization. ANLN enhances angiogenesis in OSCC and stimulates vascular branching by upregulating vascular endothelial growth factor A(VEGFA) (Wu et al., 2024). A recent study by Wu et al. showed that ANLN is a specific target gene of miR-374a-5p, and its overexpression promotes OSCC progression by escaping miR-374a-5p regulation (Wu et al., 2025). Wang et al. found that miR-497 inhibits NPC tumor progression by targeting ANLN (Wang S. et al., 2015). Furthermore, ANLN overexpression has been shown to promote the proliferation of NPC cells *in vitro*, while ANLN knockdown in xenograft models inhibits tumor growth (Zhu et al., 2025). ANLN can promote Macrophage count M2 polarization through the PTEN/PI3K/Akt signaling pathway, thereby promoting the growth of HNSCC (Zhu et al., 2025).

The above evidence indicates that ANLN has played a role in head and neck cancers, suggesting that it may serve as a new prognostic biomarker and therapeutic target.

### 4.2 The role and mechanism of ANLN in upper gastrointestinal tract cancer

ANLN is highly expressed in esophageal squamous cell carcinoma(ESCC), and its elevated expression is associated with poor prognosis in patients with ESCC (Cao et al., 2023). ANLN is also highly expressed in human gastric cancer tissues, and its expression significantly correlates with tumor size and pTNM stage.

Studies have shown that the deubiquitinase USP10 stabilizes ANLN by interacting with it and removing its K11/K63 ubiquitin chains, preventing ANLN degradation and promoting the progression of ESCC cells (Cao et al., 2023). The latest research by Cao et al. demonstrated that nuclear-localized ANLN directly interacts with the large subunit of RNA polymerase II(Pol II) through a phase separation mechanism. This interaction promotes transcriptional condensate formation, enhances Pol II aggregation initiation, and facilitates liquid-liquid phase separation of its carboxy-terminal domain, thereby regulating the expression of redox, Wnt, and differentiation-related genes. Knockdown of ANLN impairs RNA polymerase II chromatin binding and reduces enhancer-mediated transcriptional activity. Additionally, the super enhancer inhibitor THZ1 blocks gene expression by inhibiting ANLN-RNA polymerase II condensates, thereby suppressing ESCC growth (Cao et al., 2025). Su et al. (2025) found that HOXC-AS1 relieves the inhibition of ANLN by sponging miR-195-5p, thereby driving the malignant progression of esophageal cancer. This study suggests that ANLN could be a potential therapeutic target for ESCC.

### 4.3 The role and mechanism of ANLN in lung cancer

ANLN is highly expressed in lung cancer and plays a crucial role in the malignant progression of tumors through multiple pathways. Analyses of tumor mRNA expression profiles from the TCGA database and ANLN protein expression data from the HPA database revealed that ANLN expression levels in lung cancer tissues were significantly higher than those in adjacent normal tissues (Zhang L. et al., 2023). Overexpression of ANLN was significantly associated with clinical factors such as age, gender, TNM stage, and pathological grade of lung cancer patients (Wang et al., 2023). Additionally, lung cancer patients with high ANLN expression had shorter overall survival(OS), disease-free interval(DFI), and progression-free interval(PFI) compared to those with lower expression levels (Zhang L. et al., 2023).

Luo et al. established an independent prognostic model for lung adenocarcinoma(LUAD) based on two immunization-related genes, ANLN and F2. This model was capable of identifying high-risk patients, and it revealed that the immune microenvironment characteristics of high-risk patients(specifically, CD4^+^ activated T cells, Tregs, and neutrophil infiltration associated with ANLN) were significantly correlated with poor prognosis, with the model being validated in an independent dataset (Luo et al., 2020). Meanwhile, numerous bioinformatics studies and lstructions have found that ANLN is a potential biomarker related to predicting prognosis (Yi et al., 2020; Zhang L. et al., 2020), and can affect the neoplasm immune microenvironment (Shi et al., 2021; Song et al., 2021).

Studies have shown that, miR-195-5p, identified in lung cancer brain metastasis, can inhibit ANLN expression, leading to G0/G1 cell cycle arrest, promoting apoptosis, and significantly reducing the invasion and migration abilities of LUAD cells (Tomioka et al., 2025). In addition to its roles in enhancing cell proliferation, migration, and EMT, and activating pathways such as PI3K/AKT and RhoA, ANLN has also been found to promote lung cancer progression by inhibiting pyroptosis. Sheng Li et al. found that ANLN knockdown led to an increased expression of inflammation-related molecules(including caspase-1, NLRP3, cleaved-GSDMD, IL-1β, ASC, and IL-18) in tumor cell lines, suggesting that ANLN inhibits LUAD progression by activating pyroptosis (Sheng et al., 2023).

However, whether plasma-based ANLN testing can contribute to the diagnosis of lung cancer as well as the mechanisms by which ANLN influences tumor-associated immune infiltration, remain subjects requiring further investigation.

### 4.4 The role and mechanism of ANLN in breast cancer

ANLN is highly expressed in breast cancer cell lines and tissues, with its expression significantly correlated with tumor size, high tumor grade, Her2 status, and Ki-67 expression levels (O’Leary et al., 2013). High ANLN expression is strongly associated with shorter overall survival, breast cancer-specific survival, and recurrence-free survival (Magnusson et al., 2016). These findings suggest that ANLN may serve as an independent prognostic factor in breast cancer, irrespective of Ki-67 expression.

ANLN promotes breast cancer progression by enhancing cell proliferation, migration, and invasiveness while inhibiting apoptosis, and it further contributes to disease development through genetic regulation and subtype-specific functions. Studies have demonstrated that ANLN enhances the proliferation rate and colony formation of MDA-MB-231 breast cancer cells (Zhou et al., 2015). An interesting study revealed that the rs3735400 variant in the ANLN gene inhibits cell proliferation and reduces breast cancer risk in BRCA1 mutation carriers. However, overexpression of the ANLN variant decreases its nuclear localization, thereby promoting cell proliferation—providing insight into the evolutionary basis of “incomplete penetrance” in hereditary cancers (Dai et al., 2025). Bioinformatics analysis has revealed that ANLN expression correlates with immune cell infiltration and the immunosuppressive microenvironment across various breast cancer subtypes (Xiao et al., 2022). ANLN is also upregulated in triple-negative breast cancer(TNBC), where it enhances cancer stemness and promotes sphere formation *via* the TWIST1 and BMP2 signaling pathways (Maryam and Chin, 2021). Given the lack of conventional hormone receptors and HER2 expression in TNBC, traditional targeted therapies have limited efficacy. Therefore, ANLN may provide a promising therapeutic target for these patients.

### 4.5 The role and mechanism of ANLN in liver cancer

Multiple bioinformatics studies have shown that ANLN expression is closely associated with the occurrence, development, and prognosis of liver cancer (Li et al., 2022; Moghimi et al., 2024; Zhou et al., 2019). ANLN expression is significantly upregulated in hepatocellular carcinoma(HCC) tissues compared to adjacent non-tumor tissues (Lian et al., 2018). Furthermore, high ANLN expression has been linked to clinical features such as serum α-fetoprotein levels ≥400 μg/L and tumor diameter ≥8 cm, but it is not significantly correlated with age, tumor number, or differentiation grade (Zhang D. et al., 2023). Further studies have indicated that high ANLN expression is associated with poorer disease progression-free survival and serves as an independent predictor of 5-year overall survival following hepatectomy (Zhang et al., 2021).

Recent findings by Nguyen et al. demonstrated that Penfluridol upregulates NRF2, which then disrupts its binding to KEAP1, leading to NRF2 accumulation. This accumulation inhibits MYC binding to the ANLN promoter, reducing ANLN transcription and inducing G2/M phase arrest, thus decreasing colony formation in HCC cells (Nguyen et al., 2024). Moreover, nuclear ANLN expression is significantly higher in HCC metastases compared to primary HCC tumors (Zhang et al., 2021). The mechanistic role of ANLN in liver cancer is particularly evident in its involvement in metastasis-driving pathways. High ANLN expression is strongly associated with an elevated risk of bone metastasis in HCC. Mechanistically, the m6A-METTL3/YTHDF1–ANLN axis promotes HCC bone metastasis through the KIF2C/mTORC1/RANKL signaling pathway. Notably, the inhibitor DZNeP suppresses HCC bone metastasis by blocking the m6A modification of ANLN, providing a promising strategy for targeted therapy (Zheng et al., 2023).

While ANLN has emerged as a potential biomarker for hepatocellular carcinoma, its precise role in cancer treatment remains an area for further exploration.

### 4.6 The role and mechanism of ANLN in pancreatic cancer

Pancreatic cancer(PC) is one of the most malignant solid tumors, with a 5-year survival rate below 10%. Its aggressive nature, combined with difficulties in early diagnosis, presents significant challenges for clinical treatment. Previous studies have shown that ANLN expression is significantly elevated in pancreatic carcinoma tissues compared to normal pancreatic tissue and pancreatitis tissue (Olakowski et al., 2009). High ANLN expression is significantly associated with tumor size, differentiation, TNM stage, lymph node metastasis, and distant metastasis in pancreatic carcinoma, and patients with elevated ANLN expression have a poorer overall survival rate compared to those with low expression (Wang et al., 2019). Notably, Zhang et al. found that levels of exosomal ANLN, ITGA6, and KRT18 were decreased in pancreatic carcinoma patients, suggesting that the serum exosomal ANLN/ITGA6/KRT18/MMP9 RNA combination could serve as a novel non-invasive diagnostic tool for digestive system cancers (Zhang et al., 2022b).

Experimental studies have confirmed that ANLN overexpression enhances proliferation, colony formation, migration, and invasion of pancreatic carcinoma cells. The mechanism underlying this involves ANLN inhibition of miR-218-5p expression through regulation of the histone methyltransferase EZH2, thereby disinhibiting the downstream target gene LASP1 and promoting tumor cell proliferation (Wang et al., 2019). In addition, ANLN also has specific regulatory effects in pancreatic cancer. Song et al. found that ANLN overexpression increases glycolytic activity, promoting tumor cell migration and emphasizing its role in immune escape through the NF-κB signaling pathway (Song et al., 2025). As a downstream effector of the MIR600HG/hsa-miR-342-3p axis, ANLN influences tumor immune responses by regulating the expression of CCL5 and CCL14, thus altering the infiltration levels of helper T cells(Th2) and effector memory T cells(Tem) (Qian et al., 2023). Recent studies have demonstrated that HMGA2 directly regulates ANLN expression by binding to the −3,900 to −3,800 bp DNA fragment of the ANLN gene, driving tumorigenesis in pancreatic carcinoma. Together, ANLN and HMGA2 form a novel cancer-promoting axis, which could provide new therapeutic targets (Guo et al., 2020).

Although ANLN can drive the progression of PC through multiple pathways, further research is required to clarify its role in immune escape mechanisms, facilitate the development of targeted therapies, and support the clinical validation of exosomal ANLN in combination with biomarkers.

### 4.7 The role and mechanism of ANLN in urinary system cancer

ANLN is upregulated in both clear cell renal cell carcinoma(ccRCC) and urothelial carcinoma (Gao et al., 2024; Yang et al., 2023). Moreover, ANLN expression is significantly higher in metastatic ccRCC tissues compared to normal renal tissues (Wei et al., 2019). Elevated ANLN expression is significantly associated with N stage, M stage, tumor grade, clinical stage, and T stage in ccRCC (Shan et al., 2025). It is also positively correlated with high-grade tumors, advanced TNM stage, lymph node metastasis, vascular invasion, and tumor necrosis in urothelial carcinoma, with nuclear ANLN expression showing a stronger association in upper tract urothelial carcinoma(UCUT). Furthermore, higher ANLN expression correlates with worse patient prognosis in both ccRCC and urothelial carcinoma (Wei et al., 2019; Zeng et al., 2017).

ANLN promotes cell proliferation, migration, and EMT through regulation of the PI3K/Akt/mTOR and p53 signaling pathways, and further drives tumor progression by modulating the immune microenvironment. Pharmacologic inhibition of the PI3K/Akt pathway or activation of the p53 signaling pathway effectively reduces ccRCC invasiveness and reverses immunosuppression (Shan et al., 2025). ANLN expression has been positively correlated with M2 macrophage infiltration, suggesting the remodeling of the immunosuppressive microenvironment (Gao et al., 2024). Moreover, ANLN can upregulate the expression of MAPK8/MAPK9, activate phosphorylated JNK, and enhance the proliferation, migration, and invasion of bladder cancer cells while inhibiting apoptosis (Chen et al., 2023).

In conclusion, ANLN serves as a key driver in taant progression of urinary system tumors by activating the PI3K/Akt-p53 signaling pathway and reshaping the M2-type immune microenvironment. Its prognostic significance and therapeutic potential provide promising avenues for clinical intervention.

### 4.8 The role and mechanism of ANLN in colorectal cancer

ANLN is highly expressed in colorectal cancer(CRC) tissues, and its expression is positively correlated with tumor invasion and growth (Liu et al., 2022; Shi et al., 2022; Wang et al., 2016). Increased ANLN expression is an independent prognostic factor for CRC patients, with Kaplan-Meier survival analysis showing that higher ANLN levels are associated with shorter overall survival (Wang et al., 2016).

At the molecular level, in addition to the PI3K/AKT signaling pathway, ANLN promotes CRC proliferation through SP2-mediated transcriptional activation and activation of the MAPK pathway (Liu et al., 2022). However, the precise molecular mechanisms underlying ANLN activation in CRC and its carcinogenic effects have not been fully elucidated, warranting further investigation.

### 4.9 The role and mechanism of ANLN in prostatic cancer

Prostate cancer(PCa), particularly castration-resistant prostate cancer(CRPC), is a common and often fatal disease. Tamura et al. were the first to analyze gene expression profiles from 25 clinical CRPC cases and 10 hormone-sensitive prostate cancer(HSPC) cases using genome-wide cDNA microarrays combined with laser microbeam microdissection. Their study revealed that ANLN is overexpressed during the progression of hormone-refractory prostate cancer(HRPC) (Tamura et al., 2007).

Recent studies have also demonstrated that ANLN is highly expressed in CRPC tissues and is associated with more advanced clinical features, including higher T stage, N stage, Gleason score, and prostate-specific antigen(PSA) levels (Takayama et al., 2019; Yamamoto et al., 2024). Moreover, positive expression of ANLN has been shown to be a significant predictor of poor prognosis in prostate cancer patients (Yamamoto et al., 2024).

ANLN promotes the proliferation and migration of prostate cancer cells both *in vitro* and *in vivo*, and activates multiple signaling pathways to facilitate tumor development. Mechanistically, the tumor-suppressive effect of ANLN knockdown on prostatic carcinoma cell growth is partially reversed by IGF2BP1 overexpression, indicating that ANLN facilitates prostate cancer progression by stabilizing the proto-oncogene c-Myc through IGF2BP1 and activating the MAPK signaling pathway (Liu et al., 2024). Vitamin D3, a steroid hormone known for its anti-tumor properties, has been shown to influence prostate cancer development (Siddappa et al., 2023). Johnson et al. performed RNA-seq analysis on 1α, 25(OH)_2_D_3_-treated/untreated non-malignant African American prostate cells(RC-77N/E), in combination with TCGA-PRAD cohort screening. They identified ANLN as a key gene containing a vitamin D response element(VDRE), which is highly expressed in prostate carcinoma tissues. Overexpression of ANLN in prostate cancer was significantly associated with poor prognosis and increased Gleason scores in patients (Johnson et al., 2024).

These results underscore that ANLN is involved hcurrence and progression of prostate cancer and may offer new therapeutic strategies for the treatment of CRPC.

### 4.10 The role and mechanism of ANLN in cervical cancer

Analysis of the GEO database and clinical samples has demonstrated that ANLN is highly expressed in cervical cancer. Survival analysis showed that patients with low ANLN expression had significantly better survival rates compared to those with high ANLN expression (Xia et al., 2018).

Li et al. included ANLN in a six-gene risk model and verified that ANLN serves as an independent prognostic marker for cervical cancer. This model, which includes ANLN, effectively predicts the survival outcomes of cervical cancer patients, with a high degree of accuracy for 1-, 5-, 10-, and 15-year survival rates (AUC>0.7) (Li et al., 2021). Furthermore, bioinformatics analysis has suggested that ANLN may form a cell cycle regulatory network with CCNE2/CDK1/AURKA, which is associated with lymph node metastasis in cervical cancer (Ding et al., 2023). These findings suggest that ANLN plays a crucial role in the occurrence and development of cervical cancer and may serve as a novel therapeutic target for cervical cancer treatment.

### 4.11 The role and mechanism of ANLN in hematological malignancies(acute myeloid leukemia)

Unlike solid tumors, the oncogenic role of ANLN in acute myeloid leukemia(AML) arises from distinct genetic alterations. Campregher et al. identified a t(7; 12)(p15; p13) chromosomal translocation that generates an ETV6-ANLN fusion gene, resulting in high expression of the ETV6-ANLN transcript in both myeloid and lymphoid lineages, thereby implicating ANLN in the pathogenesis of hematologic malignancies (Campregher et al., 2015). This discovery provides the first direct evidence linking ANLN abnormalities to the onset of blood cancers and highlights a mechanistically distinct mode of ANLN activation compared with solid tumors.

In conclusion, ANLN exhibits significant carcinogenic effects across various tumor types. Further investigation into its expression patterns, biological functions, and molecular mechanisms in different cancers will enhance our understanding of its universal role in tumor progression and facilitate the clinical validation of its prognostic and targeted therapeutic value.

## 5 Tumor treatment resistance mediated by ANLN and targeted strategies

### 5.1 Tumor treatment resistance mediated by ANLN and reversal of tumor resistance

**FIGURE 3:**
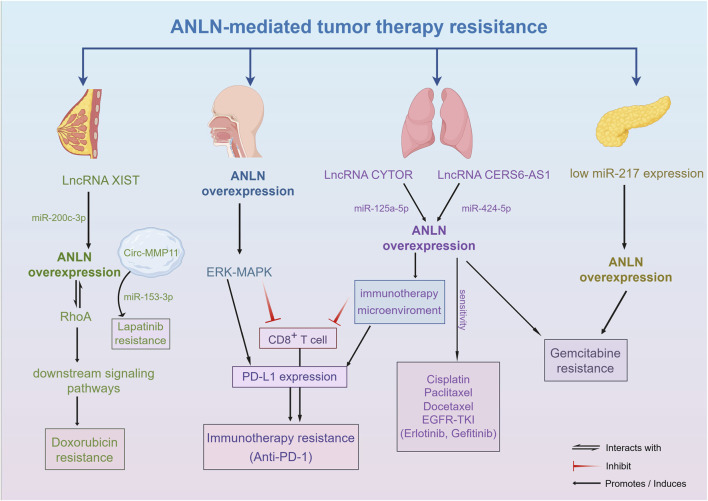
The molecular mechanism of tumor treatment resistance mediated by ANLN. Aberrant expression of ANLN contributes to resistance against chemotherapy, targeted therapy, and immunotherapy by modulating cell cycle control, DNA repair, and apoptotic signaling.

Treatment-related drug resistance and the resultant tumor progression are major contributors to the poor prognosis observed in various cancers. Drug-tolerant tumors often exhibit mutations in specific oncogenes or tumor suppressor genes, or an aberrant overexpression of key regulatory genes. ANLN is abnormally overexpressed in a variety of solid tumors, and its expression level is significantly associated with clinical drug resistance in chemotherapy, targeted therapy, and immunotherapy (Figure 3; Table 1).

Studies have shown that, in breast cancer patients, the disease-free survival(DFS) of the low ANLN expression group receiving anthracycline-based chemotherapy was significantly better than that of the high expression group (Wang et al., 2017). Targeting ANLN directly, Doxorubicin-resistant breast cancer cells were sensitized to the drug, resulting in significantly reduced cell viability and enhanced apoptosis. This may occur through ANLN’s interaction with RhoA, thereby promoting resistance of breast cancer cells to Doxorubicin (Wang F. et al., 2020). Furthermore, ANLN expression is positively correlated with Gemcitabine resistance, and the mechanism may involve miR-217, which promotes S-phase arrest, inhibits pancreatic cancer cell proliferation, and enhances Gemcitabine sensitivity by downregulating ANLN expression (Panebianco et al., 2021).

In addition, various non-coding RNAs have been found to promote chemotherapy resistance by regulating ANLN expression, with resistance mechanisms confirmed in drugs such as Paclitaxel, Gemcitabine, Doxorubicin, Imatinib, and Lapatinib (Chen et al., 2025; Yang et al., 2022). CYTOR, an oncogenic long non-coding RNA(LncRNA), functions as a competitive endogenous RNA(ceRNA) to upregulate ANLN expression by adsorbing miR-125a-5p, inhibiting its degradation, and thereby promoting Gemcitabine resistance in LUAD (Cao et al., 2024). XIST, another LncRNA, also promotes chemotherapy resistance in breast cancer cells to Doxorubicin by competitively binding miR-200c-3p and relieving the inhibition of ANLN (Zhang M. et al., 2020). Circular RNA(CircRNA) Circ-MMP11 contributes to metastasis *via* exosomes in breast cancer cells, adsorbing miR-153-3p to upregulate ANLN expression, thereby promoting Lapatinib resistance (Wu et al., 2021).

In the context of immunotherapy, Wang et al. found that in head and neck squamous cell carcinoma, highly expressed ANLN activates the ERK-MAPK signaling pathway, significantly upregulates PD-L1 expression, inhibits CD8^+^ T cell activation, and reduces the efficacy of anti-PD-1 monoclonal antibodies (Wang et al., 2025). Ting et al. showed that ANLN is a potential target gene of miR-424-5p, and LncRNA CERS6-AS1 upregulates ANLN by sponging miR-424-5p. Moreover, their analysis suggested that ANLN affects immune cell infiltration, particularly increasing the infiltration of resting anti-tumor immune cells and immunosuppressive cells, and correlates with a higher tumor mutation burden. Interestingly, further studies found that high ANLN expression increases lung cancer cells’ sensitivity to Cisplatin, Paclitaxel, Docetaxel, and EGFR-TKIs(Erlotinib, Gefitinib) (Ting et al., 2024). In Paclitaxel-resistant LUAD patients, high ANLN expression is closely associated with an immunosuppressive microenvironment, including upregulation of PD-L1 and exhaustion of CD8^+^ T cells.

### 5.2 Targeting ANLN for tumor treatment

**FIGURE 4:**
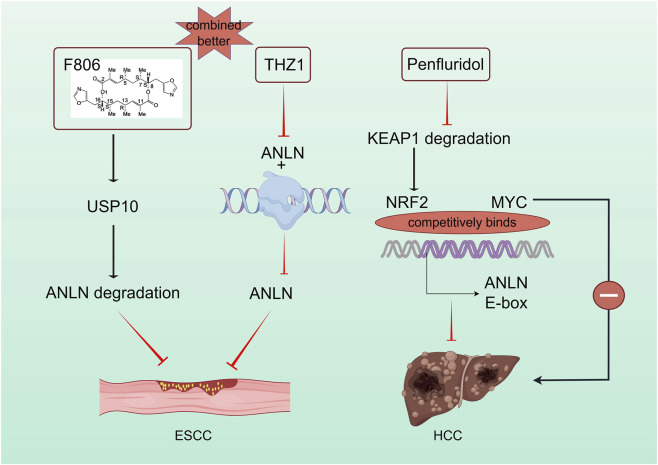
Targeting ANLN for the treatment of esophageal cancer and liver cancer. Inhibitors suppressing ANLN through direct or indirect mechanisms offer potential for ANLN-targeted tumor therapy.

In recent years, targeting ANLN has shown significant potential in tumor therapy, providing novel strategies for the development of precise therapeutic approaches. Nguyen et al. identified Penfluridol as the first ANLN inhibitor, demonstrating its efficacy in liver carcinoma. Penfluridol induces NRF2 protein accumulation by inhibiting KEAP1-mediated ubiquitination, thereby blocking MYC-mediated ANLN transcriptional activation. This inhibition of ANLN progression represents a breakthrough in developing targeted drugs for ANLN (Nguyen et al., 2024; Figure 4).

Additionally, siRNA-based ANLN-targeted strategies have shown promising therapeutic effects in liver and cervical cancer. Studies have confirmed that lipid nanoparticles loaded with ANLN small interfering RNA(siRNA) inhibit liver cancer cell proliferation without impairing the regenerative capacity of normal liver cells. Furthermore, N-acetylgalactosamine(GalNAc)-conjugated siANLN exhibits liver-targeting specificity, offering a novel precision therapy for liver cancer prevention and treatment (Zhang et al., 2018).

Recently, Maggiore et al. used GalNAc-conjugated siRNA technology to simultaneously target five genes, including ANLN, in four liver cancer models. Their study confirmed that hepatocellular carcinoma development is driven by these oncogenes, with ANLN playing a particularly crucial role. Using the Cre-lox system, they showed that ANLN silencing alone could improve histological features of metabolic dysfunction-associated steatohepatitis(MASH) and inhibit HCC progression (Maggiore et al., 2025).

Pan et al. constructed ANLN-targeting siRNA and found that the cell transfection efficiency in the UTMD-siANLN group was higher than in the liposome transfection group. The UTMD-siANLN group exhibited stronger inhibition of the malignant phenotype in cervical carcinoma cells, suggesting that UTMD-siANLN may be an effective strategy for improving the prognosis and treatment of cervical cancer in clinical practice (Pan et al., 2022).

ANLN also plays a key role in other drug-targeting strategies. The macrolide compound FW-04-806(F806), a potential treatment for ESCC, inhibits ESCC cell division by targeting USP10 and promoting ANLN degradation. F806 selectively targets USP10 without affecting Cdh1-ANLN binding, altering the USP10-Cdh1-ANLN complex balance. Additionally, the super-enhancer inhibitor THZ1 specifically inhibits ANLN-Pol II aggregation, suppressing ESCC development, while the combined effect of F806 and THZ1 exceeds their individual effects (Cao et al., 2025; Figure 4).

Lastly, recent studies identified the cholesterol–TAZ–TEAD2–ANLN/KIF23 axis in hepatocellular carcinoma drug resistance. Dysregulated cholesterol metabolism activates TAZ, a downstream effector of the Hippo pathway, leading to its interaction with the TEAD2 transcription factor. This complex upregulates ANLN and KIF23 expression, promoting tumor proliferation while remodeling the immunosuppressive microenvironment. Targeting this pathway with pan-TEAD inhibitors or statins like simvastatin, in combination with sorafenib or PD-1 monoclonal antibodies, can suppress tumor growth and reverse immune evasion (Saito et al., 2023).

## 6 The limitations of the current study and future prospects

This review systematically explores the crucial role of ANLN in various cancers, focusing on its involvement in tumorigenesis, progression, and drug resistance. As an important molecule involved in cell division and cytoskeletal regulation, ANLN plays a critical role in several cellular processes that contribute to cancer development. Despite extensive research showing ANLN overexpression in various malignancies, its precise molecular mechanisms remain incompletely understood and require further investigation.

Firstly, although numerous studies have demonstrated that ANLN overexpression promotes tumor initiation and progression through multiple regulatory mechanisms, the core nature of its oncogenic role remains controversial. On one hand, functional gain- and loss-of-function studies have shown that ANLN can actively drive cell-cycle progression, inhibit apoptosis, enhance invasion, and exert pro-tumorigenic effects by activating key pathways such as PI3K/AKT and RhoA, supporting the notion that overexpressed ANLN acts as a true “driver.” On the other hand, as an essential mediator of cytokinesis, ANLN expression may passively increase in parallel with elevated proliferative activity. Thus, its upregulation in highly proliferative tumors may simply represent a “proliferation bystander.” To resolve this ambiguity, future studies should employ more precise experimental systems—such as conditional gene knockout and inducible expression models—to definitively establish causality.

Secondly, although ANLN promotes tumor proliferation through shared mechanisms—including regulation of the cell cycle, cytoskeletal dynamics, and EMT, as well as participation in several canonical oncogenic pathways—its regulatory networks and functional emphasis differ substantially across cancer types. For example, in esophageal squamous cell carcinoma, ANLN regulates transcription through nuclear phase separation; in liver cancer, its expression is finely tuned by m6A modifications and plays a specific role in driving bone metastasis; whereas in AML, unique gene-fusion events underlie its activation. This mechanistic heterogeneity suggests that ANLN may play a more central or specialized role in certain tumor contexts. However, our current understanding of the molecular basis underlying this tissue specificity remains limited. Future studies should incorporate cross-cancer comparative analyses to construct a comprehensive “ANLN regulatory atlas” and to delineate its cancer-type-specific and universal mechanisms.

Furthermore, ANLN’s role in drug resistance makes it a key target in cancer therapy. While studies have shown that ANLN plays an important role in chemotherapy, targeted therapy, and immunotherapy resistance, the mechanisms remain complex and multifactorial. Research suggests that ANLN may promote cancer cell drug resistance by regulating EMT and RhoA-associated pathways. Gaining a deeper understanding of how ANLN regulates EMT and its role in resistance could provide new insights into overcoming resistance and improving therapeutic outcomes. Meanwhile, ANLN’s role in the immune microenvironment has also gained increasing attention. Current research indicates that ANLN may play a significant role in immune therapy by regulating immune cell infiltration, PD-L1 expression, and immune evasion mechanisms. Further studies will help reveal how ANLN affects the efficacy of immunotherapy, particularly in how immune evasion mechanisms in the tumor microenvironment are linked to ANLN expression.

Additionally, while ANLN’s potential as a biomarker has been validated in several studies, its clinical application still faces challenges. Currently, ANLN detection primarily relies on immunohistochemistry and qPCR methods. However, the potential for non-invasive detection of ANLN in peripheral blood has not received sufficient attention. Future research should focus on developing liquid biopsy-based methods for ANLN detection to enable early cancer detection and progression monitoring.

Finally, although targeted ANLN therapies have shown promise in preclinical models, specific inhibitors of ANLN are still in their infancy. Penfluridol, the first identified ANLN inhibitor, has demonstrated efficacy in liver cancer by inhibiting ANLN transcription. However, further research is required to develop small molecule inhibitors or RNA interference technologies that can effectively target ANLN. Combining ANLN-targeted therapies with conventional treatments may offer synergistic effects, improving the therapeutic response in tumors with high ANLN expression and treatment resistance.

## 7 Conclusion

In conclusion, ANLN is a key regulatory molecule in tumor progression and drug resistance in various cancers. ANLN is overexpressed in various cancer types and is closely associated with tumor proliferation, migration, invasion, and EMT, and it is linked to poor prognosis in cancer patients. Current studies indicate that ANLN promotes tumor progression by regulating the cell cycle, cytoskeletal dynamics, and multiple oncogenic pathways, including RhoA and PI3K/AKT. ANLN is also implicated in the development of resistance to chemotherapy, targeted therapy, and immunotherapy.

Although ANLN shows substantial potential as a biomarker and therapeutic target, several limitations remain. The causal relationship between ANLN dysregulation and tumor initiation is not fully established, the cancer-specific regulatory networks governing its activity are incompletely understood, and effective, highly specific ANLN inhibitors are still lacking. Future research should aim to elucidate the molecular mechanisms underlying ANLN-driven tumorigenesis and therapy resistance. Integrating multi-omics approaches, cross-cancer comparative analyses, and RNA interference or gene-editing technologies may accelerate the clinical translation of ANLN-targeted therapies. In addition, the development of non-invasive detection strategies, such as ANLN-based liquid biopsy assays, may provide new opportunities for early diagnosis and treatment monitoring.

Overall, ANLN represents a compelling therapeutic target in oncology. Its involvement in tumor progression, drug resistance, and immune regulation underscores its promise as a future research focus. A deeper understanding of its molecular functions is expected to yield breakthroughs in overcoming therapeutic resistance, improving patient prognosis, and advancing the development of novel targeted treatment strategies.
